# Editorial: Stroke in Elderly: Current Status and Future Directions

**DOI:** 10.3389/fneur.2019.00177

**Published:** 2019-03-01

**Authors:** Muhib Khan, Brian Silver

**Affiliations:** ^1^Division of Clinical Neuroscience, Michigan State University, East Lansing, MI, United States; ^2^UMass Memorial Medical Center, Worcester, MA, United States

**Keywords:** stroke, elderly, stroke recovery, thrombectomy, carotid disease

The worldwide elderly population is rapidly growing^1^. Stroke incidence doubles in octogenarians (1). In the US, the number of people older than 85 years, who currently constitute 2% of the total population, will double by 2060 (2). Therefore, the absolute number of strokes is expected to significantly increase over the course of the next century.

It is therefore important for physicians to understand the etiologies of stroke in this population, minimize risk factors for stroke, manage acute stroke in effective manner, and focus on rehabilitation. In this Research Topic, we focused on research pertaining to elderly stroke.

**Prevention:** Heo and Bushnell address the topic of carotid disease in the elderly. They highlight the differences in post-procedural stroke and mortality between carotid stenting and carotid endarterectomy among elderly patients. In addition to age, other predictors of outcome include gender, comorbid conditions, and symptomatic status. Wang et al. focus on asymptomatic carotid stenosis. Functional MRI and cognitive testing suggest that carotid stenting for asymptomatic carotid stenosis can improve cognition by increasing perfusion in the left frontal gyrus and connectivity in the posterior cingulate cortex. Cognitive outcomes are an important aspect of care in the elderly and this article provides insight into the role of stenting in improving cognition for the elderly patients.

**Blood Pressure:** Geng et al. highlight the impact of blood pressure variability in the subacute phase of stroke on long term cognitive outcomes in the elderly. Blood pressure management for the elderly has been a source of debate and this article provides valuable information on how better blood pressure management impacts cognition in the elderly post-stroke patients.

**Acute Stroke Treatment:** Jayaraman and McTaggart discuss the outcomes of thrombectomy for large vessel occlusion related stroke in the elderly. They provide a detailed review of recent trials in the context of outcomes for elderly undergoing thrombectomy and the need for further studies. Peng et al. evaluate the impact of apolipoprotein E ε4 on imaging and clinical markers in patients with subarachnoid hemorrhage. Patients with the apolipoprotein E ε4 allele were found to have raised intracranial pressure and decreased perfusion in the white matter region after subarachnoid hemorrhage. Apolipoprotein E is a major focus for the elderly and this article ties it to subarachnoid hemorrhage outcomes. Khan et al. evaluate the impact of specific infarct location in distal middle cerebral artery occlusion stroke on outcome. They demonstrate specific infarct locations to be predictive of outcomes with no impact of laterality. Prognostication post stroke in the elderly population is an important aspect of care for the elderly and location of infarct provides valuable insight into prognosis.

**Recovery:** Xing et al. assess the impact of white matter connectivity on post stroke aphasia utilizing functional magnetic resonance imaging (fMRI). They note a role of temporal lobe pathways in word-level and sentence-level comprehension pertaining to post-stroke aphasia. Wall et al. also evaluate post stroke aphasia in the context of cognition. They emphasize the need for better strategies to test cognition in patients with aphasia after stroke. Grech et al. tackle a difficult issue of post-stroke spatial neglect. They suggest incorporating Mobility Assessment Course (MAC) testing when evaluating spatial neglect. Marei et al. discuss the use of stem cells for enhancing recovery after stroke and highlight the need for further large clinical trials. The articles provide impact information of recovery pertaining to the elderly which will enable us in prognostication as well as designing better intervention strategies in the post-acute phase.

In summary, elderly stroke patients are a unique group requiring a multifaceted approach incorporating prevention, acute treatment and focus on recovery. In view of the growing elderly population, there is a significant need for further research to improve outcomes for these patients.

## Author Contributions

All authors listed have made a substantial, direct and intellectual contribution to the work, and approved it for publication.

### Conflict of Interest Statement

The authors declare that the research was conducted in the absence of any commercial or financial relationships that could be construed as a potential conflict of interest.

## Footnotes

^1^https://population.un.org/wpp/Publications/Files/WPP2017_Wallchart.pdf
